# In-field nucleic acid testing for porcine epidemic diarrhea virus with lateral flow immunoassay

**DOI:** 10.3389/fvets.2025.1535605

**Published:** 2025-05-21

**Authors:** Wencong Chen, Jiahao Chen, Danni Ye, Xueyan Ai, Jiayi Luo, Chenxi Fang, Meihua Xiang, Maopeng Wang

**Affiliations:** ^1^Wenzhou Key Laboratory for Virology and Immunology, Institute of Virology, Wenzhou University, Chashan University Town, Wenzhou, China; ^2^Research and Development Department, Hangzhou Goodhere Biotechnology Co., Ltd., Hangzhou, China

**Keywords:** PEDV, M gene, in-field detection, lateral flow immunoassay, microfluidic chip

## Abstract

Porcine epidemic diarrhea is a significant disease that damages the global pig industry. The delay between infection and vaccination allows for a 30–70% mortality rate among piglets. Early virus detection is a more effective disease management strategy for pigs. Unfortunately, most methods rely heavily on skilled personnel and sophisticated equipment, which is not ideal in scenarios requiring cost reduction and efficiency. To address these challenges, our study based on one-step RT-PCR and lateral flow strips developed a reliable, field-deployable, and low-cost nucleic acid detection method for PEDV. The whole process takes about 45 min and the testing cost of each sample is low to $0.3. Moreover, this method is simple to operate, it is possible to complete the entire operation without training. Our clinical application demonstrates a robust diagnostic tool for detecting PEDV in field conditions, thereby providing more time for PEDV epidemic emergency response.

## 1 Introduction

Porcine Epidemic Diarrhea Virus (PEDV) is an enveloped, single-stranded, positive-sense RNA virus of the coronavirus family (1). It is a highly contagious viral disease firstly observed in farm pigs in the UK in the 1970s. Since then, PEDV has spread in Europe and Asia, becoming an epidemic in many countries (2, 3). Generally, in susceptible herds, PEDV is characterized by a rapid onset of watery diarrhea and vomiting in pigs of all ages, with a mortality rate of nearly 100% in suckling pigs (4). This has caused devastating economic losses to the pig industry. Since it is almost impossible to eradicate PEDV soon, it is important to prevent or control the damage caused by this disease by monitoring viral nucleic acid in pig populations (5). Currently, various detection methods for PEDV have been reported, such as virus isolation (6), direct electron microscopy (EM) (7), Enzyme-Linked Immunosorbent Assays (ELISA) (8), and real-time PCR (9). However, these technologies have some limitations that they require expensive equipment, time, and skilled personnel (10). To shorten the molecular diagnosis time, we have developed an in-field, point-of-care nucleic acid detection (POC-NAD) method based on one-step RT-PCR and lateral flow strips to minimize the occurrence of false positives for airborne viruses such as PEDV (Supplementary material Figure 1).

## 2 Materials and methods

### 2.1 DNA/RNA extraction

Viral nucleic acids were extracted using a magnetic bead-based DNA/RNA co-extraction kit (Niu-gene, M063-100). Briefly, the sample was lysed with proteinase K and buffer (VLr), then incubated with magnetic beads for 5 minutes to bind nucleic acids. After immobilization on a magnetic rack, the beads were briefly dried (1 min), and nucleic acids were eluted into a new tube for downstream use.

### 2.2 Viral genomes

Viral genomes (DNA or RNAs) used for a one-step PCR assay were stored in the laboratory, including African swine fever virus (ASFV), Porcine epidemic diarrhea virus (PEDV), porcine rotavirus (PRoV), Porcine Reproductive and Respiratory Syndrome (PRRSV), Porcine delta coronavirus (PDCoV), and Transmissible gastroenteritis virus (TGEV).

### 2.3 Positive reference Preparation

We amplified the PEDV M gene (CV777 strain) using specific primers. The PCR products were purified (DC301-01, Vazyme) and cloned into a pEasy-Blunt vector (CB101-01, TransGene Biotech). The recombinant plasmid was transformed into Trans-T1 competent cells (CD501-02, TransGene Biotech). In vitro transcription was performed using the T7 High Yield RNA Transcription Kit (Vazyme, Supplementary materials Figure 2), followed by RNA purification via the Trizol method. RNA concentration was measured using a NanoDrop 2000 spectrophotometer (Thermo Fisher)

### 2.4 In-field nucleic acid detection method

In-field nucleic acid detection method uses the portable PCR system and microfluidic chip (from Beijing Origin Biotech) for amplification. Its integrated design ensures reliable performance even in challenging field conditions. The one-step RT-PCR combines reverse transcription and amplification in a single reaction to reduce contamination risks and false positives. The reaction mix includes reverse transcriptase, DNA polymerase, UDG enzyme, RNase inhibitor, buffer (with Mg2+, dUTP, primers), and template RNA. All enzymes and reagents supplied by Fapon Biotech. The full thermal cycling process takes 47 minutes.

### 2.5 Specificity of in-field nucleic acid detection technology

The specificity of in-field nucleic acid detection was assessed the DNA genome (ASFV), as well as the RNA genomes (PDCoV, RoV, TGEV, and PRRSV) as sample templates. And the PEDV Positive reference was performed as the positive control and RNase-free water was used as the negative control.

### 2.6 Sensitivity of in-field nucleic acid detection technology

The PEDV-positive reference was used as a template, serial two-fold dilutions were prepared with nuclease-free water, achieving a template range of 7300 copies/rxn to 57 copies/rxn. The limit of detection (LoD) was determined by testing the two-fold diluted RNA template in 20 replicates, with nuclease-free water serving as the no-template control.

A control line was established to confirm assay validity, and all test strips were imaged within 1 minute using a standardized protocol. Quantitative analysis was performed using ImageJ software, revealing a statistically significant positive correlation between the optical density of the colloidal gold detection system and the viral nucleic acid copy number.

Concentration and copy number conversion formula:

Kappa formula (PA represents the probability of observing agreement and PE represents the probability of expecting agreement):

$$Kappa=\frac{PA-PE}{1-PE}$$

## 3 Results

**Figure 1:**
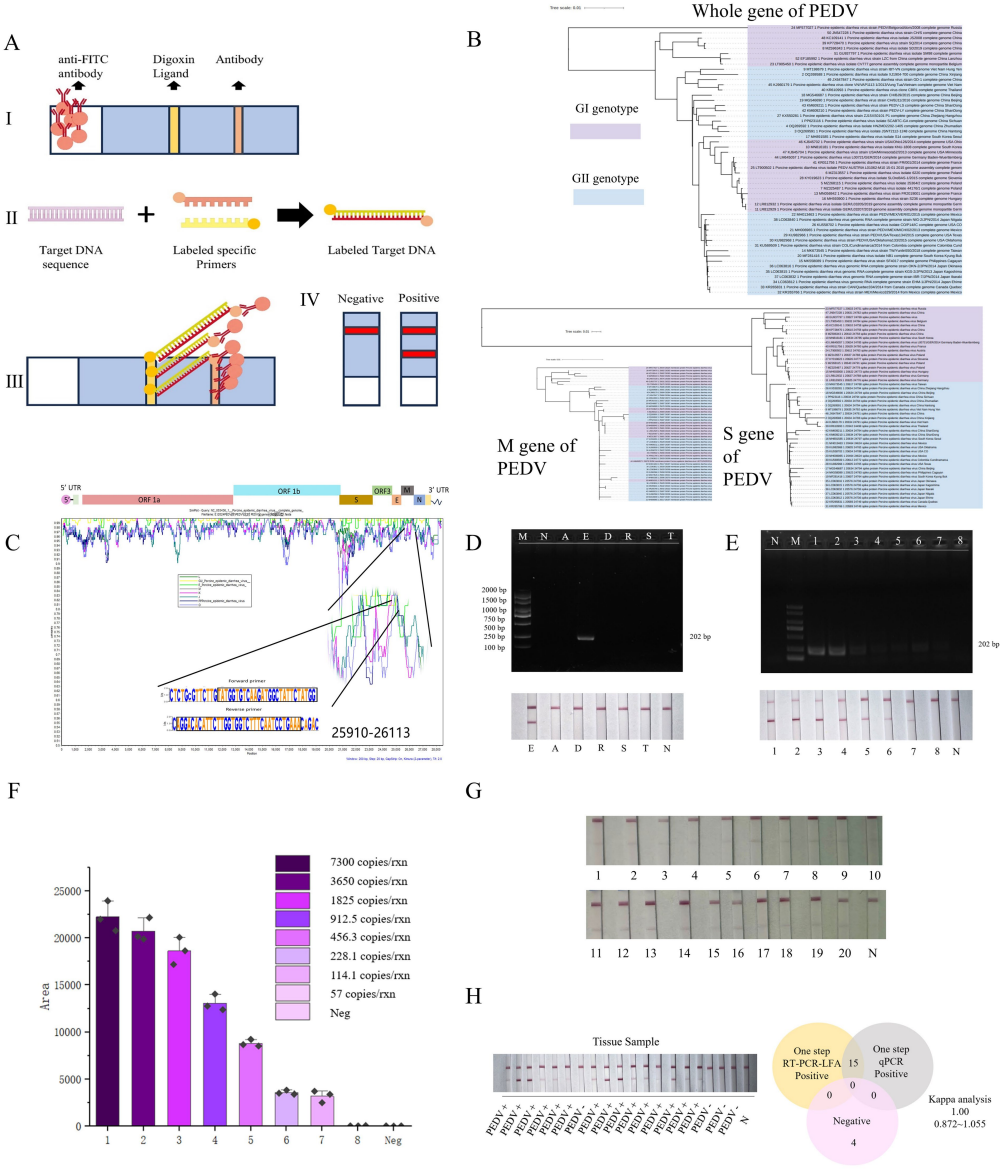
The PEDV POC-NAD technique. **(A)** Double antigen sandwich colloidal gold immunochromatography. I: Colloidal gold-anti-FAM antibody (mouse anti-FAM) was placed on the binding pad. The test (T) line of anti-Digoxin antibody was fixed as the detection line. A fixed sheep anti-mouse polyclonal antibody served as (control) C line, which can combine mouse antibody; II: In the presence of the target sequence, the amplified product, marked with Digoxin and FAM, can be generated using specific primers; III: In the presence of the target product, a “colloidal gold – anti-FAM antibody – target product – anti-Digoxin antibody structure” was formed at the T-line to make the T-line color. At the position of line C, “Colloidal gold – anti-FAM antibody – sheep anti-mouse polyclonal antibody” was formed to make line C color; IV: Negative and positive results. **(B)** Phylogenetic tree based on 50 complete genomic, M gene, and S gene sequences of the PED virus. **(C)** The similarity of four hundred and ninety-nine complete genomic sequences was shown with WebLogo and demonstrated by using Simplot v 3.5.1 with NC003436 as the query sequence. **(D)** Results of the specificity of P-M in 1% (w/v) agarose gel electrophoresis and results on lateral flow assay. E: PEDV; D: PDCoV; R: PRoV; S: PRRSV; T: TGEV; A: ASFV; N: Negative control; M: DL2000 Marker. **(E)** Results of the Sensitivity of P-M-X in 1% (w/v) agarose gel electrophoresis and results on lateral flow assay; 1–8: 7300 copies/rxn, 3,650 copies/rxn, 1825 copies/rxn, 912.5 copies/rxn, 456.3 copies/rxn, 228.1 copies/rxn, 114.1 copies/rxn, 57 copies/rxn; Neg represents the negative control. **(F)** The visual outcomes of various concentrations were subjected to grayscale analysis, an initial quantitative analysis can be conducted by using this method. **(G)** The LOD is defined as the lowest concentration at which 95% of positive samples are detected, we repeated twenty times. **(H)** The results of clinical sample detection with POC-NAD and coincidence rate of conventional RT-qPCR and POC-NAD with Kappa analysis.

The genome sequence of PEDV contains a 5’ end cap, a 5’ untranslated region (UTR), 7 open reading frames, a 3’ translation region (UTR), and a 3’ end of adenosine acidification (poly-A) tail (11). According to previous genotype classification, the complete genome and S gene are proper for viral evolutionary analysis (12). Therefore,we constructed maximum likelihood trees using PEDV whole-genome, S gene, and M gene sequences from global isolates in the NCBI database (Supplementary Table 1). The M gene-based tree showed the smallest genetic distance and lowest mutation rate compared to those based on the S gene or whole genome (Figure 1B; Supplementary Data Sheet 1–3) that its explains the indispensable role in the viral replication cycle (13). Therefore, the PEDV M protein could be a promising candidate for developing specific antiviral drugs and as a target for vaccine or primer design (14). So, we designed a pair of specific primers for viral diagnosis according to their conserved sequences (Figure 1C). Primers were synthesized by Shanghai Sangon Bioengineering Co., Ltd., China (Table 1). For visual detection, we employed a colloidal gold-based double-antigen sandwich assay, with specific antibodies immobilized on the binding pad, test line, and control line. The primers were labeled at their 5’ends. (Figure 1A).

**Table 1:** Primers.

Primer name	Primer sequence 5’-3′
P-MF-X	5´Dig -TATGGTGTCAAGATGGCTATTCTATGG
P-MR-X	5´6-FAM-AAAGACCACCAAGAATGTGTCCT

Given that other viruses could cause similar diarrhea symptoms as PEDV, it is important to differentiate PEDV from other viruses. The specificity of In-field nucleic acid detection method was detected by DNA virus as Africa swine fever virus (ASFV), and RNA viruses as PRRSV, PDCoV, PRoV, and TGEV. The results indicated that PEDV could be detected with strong signals, distinguishing it from other viruses (Figure 1D). Then, we assessed the sensitivity of In-field nucleic acid detection method using various template concentrations (Figures 1E,F). Results indicated our method could detect concentrations down to 114.1 copies/rxn (Figure 1G). In addition, the total RNA was extracted from PEDV-infected and uninfected clinical samples as a detection template (Supplementary material Figure 2). Then we compared our established method with a commercial RT-qPCR Nucleic Acid Test Kit for PEDV (GM Biotech, GM11011. And the results from commercial kit seen in Supplementary Table 3). The results revealed that both methods exhibited same results (Figure 1H). By contrast, our method offered the advantages of reading out result faster and operating more friendly.

## 4 Discussion

The PEDV disease can occur all year round, but it occurs frequently in spring and winter (3). The transmission is mainly through direct or indirect fecal-oral pathway, but the aerosol transmission in the middle and long distance also cannot be ignored (15). Thus, rapid detection of PEDV infection is crucial for current surveillance programs. Recently, the real-time RT-PCR is preferred because the assay can be used as a high throughput test system to detect PEDV genomic template during the acute phase of the infection or pre-seroconversion. However, RT-qPCR is impractical in remote areas or pig farms lacking expensive equipment like PCR instruments. Therefore, researchers have proposed numerous methods for the real-time diagnosis of PEDV. Under experimental conditions, the fluorescence-based lateral flow immunoassay for detecting PEDV antigens has demonstrated a lower detection limit of no less than 5.9×10^2^ TCID_50_ (16). However, the “reproducibility crisis” of antibodies can lead to cross-reactivity or other non-specific bindings, which may compromise the accuracy of the assay (17). In recent years, real-time diagnostic methods for PEDV nucleic acids have primarily been developed based on isothermal amplification technologies such as RPA and LAMP. Remarkable results have been achieved by incorporating SYBR Green I fluorescent dye into the LAMP system, establishing a diagnostic method with a sensitivity of 0.0001 ng/μL (18). Despite showing good specificity under laboratory conditions, it has been reported that the strand-displacing activity of Bst DNA polymerase could cause non-specific binding of primers at low concentrations or with complex templates, leading to false-positive results (19). Consequently, such methods still require further validation in field applications.

In this study, we established a In-field nucleic acid detection method based on a one-step RT-PCR method, lateral flow immunoassay technology, and microfluidic technology. Compared with other methods, our detection method is more portable, requires fewer pieces of equipment and reagents, and has lower demands for transportation and storage, making it more suitable for on-site detection. Additionally, it is cost-effective, with an average cost per reaction under $0.3, and time-efficient, completing the process from extraction to determination in under an hour. Moreover, In-field nucleic acid detection method has high sensitivity down to 114.1 copies/reaction and good specificity. In-field nucleic acid detection method has demonstrated no cross-reactivity with PDCoV, TGEV, ASFV, PRRSV, or PRoV. Regarding the limitations of In-field nucleic acid detection method, one significant drawback is the limited number of samples that can be processed in each run. Currently, each run accommodates only four samples, including the negative control. In the future, it is crucial to consider increasing the number of samples that can be analyzed per run to improve efficiency and scalability. In conclusion, this diagnostic tool is suitable for real-time nucleic acid testing in field environment and offers reliable PEDV diagnostic capability for viral endemic regions.

## Data Availability

The original contributions presented in the study are included in the article/Supplementary material, further inquiries can be directed to the corresponding author.
