# Supplementary figures and images for: In-field nucleic acid testing for porcine epidemic diarrhea virus with lateral flow immunoassay
Source: Front Vet Sci. 2025 May 21;12:1535605. doi: 10.3389/fvets.2025.1535605 (PMC12135921; doi:10.3389/fvets.2025.1535605)

Tree scale: 0.01

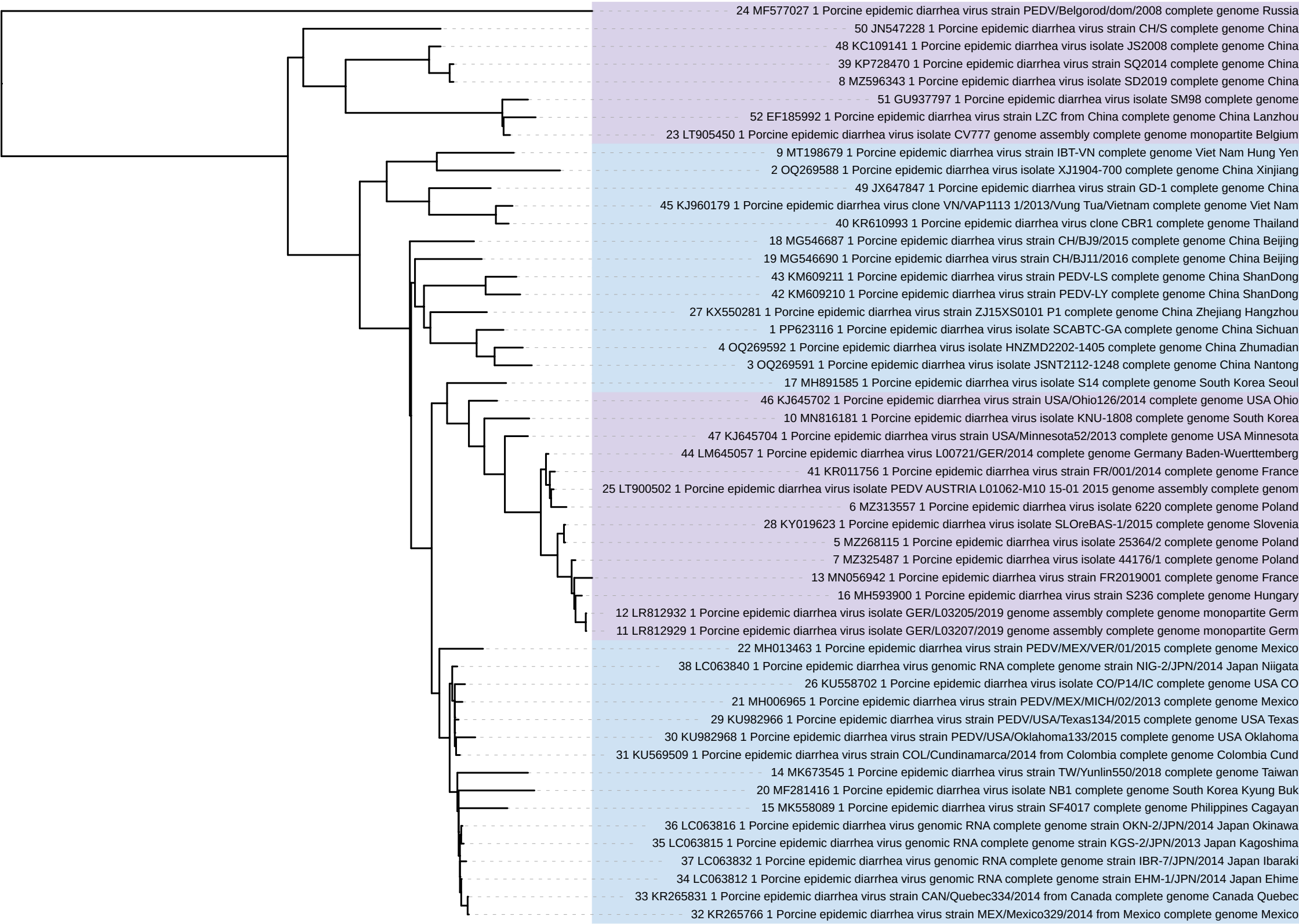

Supplement: Supplementary file 1 [file Data_Sheet_1.pdf]

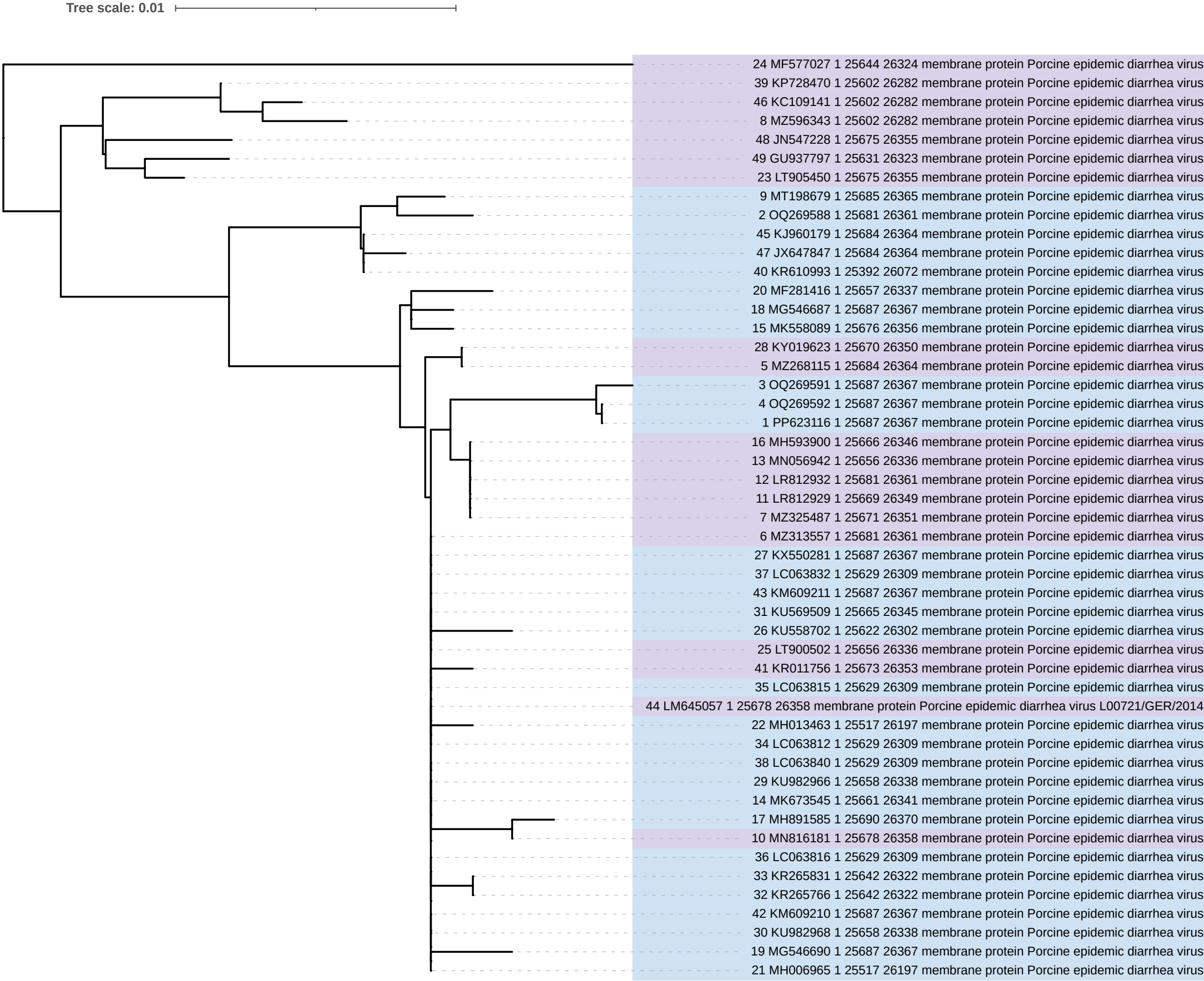

Supplement: Supplementary file 2 [file Data_Sheet_2.pdf]

Tree scale: 0.01

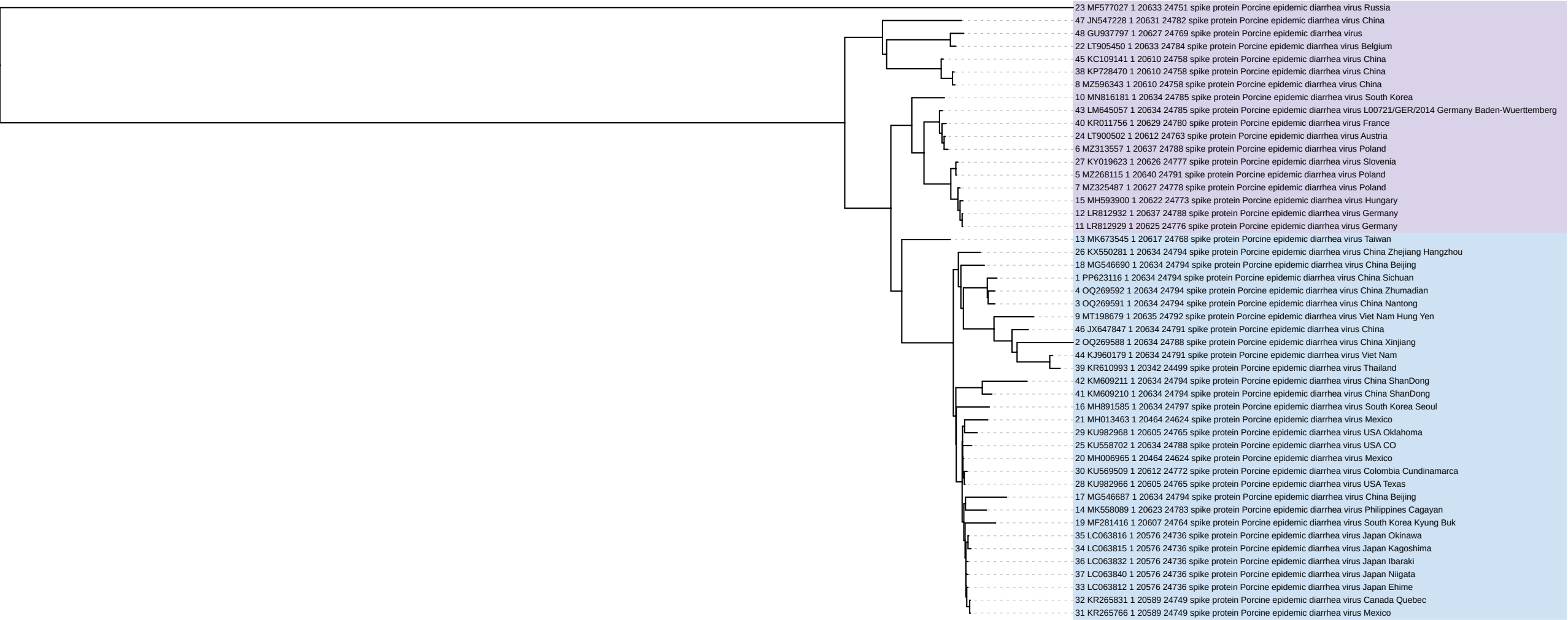

Supplement: Supplementary file 3 [file Data_Sheet_3.pdf]
