# Supplementary material for: In-field nucleic acid testing for porcine epidemic diarrhea virus with lateral flow immunoassay
Source: Front Vet Sci. 2025 May 21;12:1535605. doi: 10.3389/fvets.2025.1535605 (PMC12135921; doi:10.3389/fvets.2025.1535605)
Supplement: Supplementary file 4 [file Table_1.docx]

Supplement Table 1 complete genome, S and M genes sequences of PEDV from different countries in the NCBI genome database

| **M gene** | **Complete gene** | **S gene** |
| --- | --- | --- |
| PP623116 | PP623116 | PP623116 |
| OQ269588 | OQ269588 | OQ269588 |
| OQ269591 | OQ269591 | OQ269591 |
| OQ269592 | OQ269592 | OQ269592 |
| MZ268115 | MZ268115 | MZ268115 |
| MZ313557 | MZ313557 | MZ313557 |
| MZ325487 | MZ325487 | MZ325487 |
| MZ596343 | MZ596343 | MZ596343 |
| MT198679 | MT198679 | MT198679 |
| MN816181 | MN816181 | MN816181 |
| LR812929 | LR812929 | LR812929 |
| LR812932 | LR812932 | LR812932 |
| MN056942 | MN056942 | MK673545 |
| MK673545 | MK673545 | MK558089 |
| MK558089 | MK558089 | MH593900 |
| MH593900 | MH593900 | MH891585 |
| MH891585 | MH891585 |  |
| MG546687 | MG546687 | MG546687 |
| MG546690 | MG546690 | MG546690 |
| MF281416 | MF281416 | MF281416 |
| MH006965 | MH006965 | MH006965 |
| MH013463 | MH013463 | MH013463 |
| LT905450 | LT905450 | LT905450 |
| MF577027 | MF577027 | MF577027 |
| LT900502 | LT900502 | LT900502 |
| KU558702 | KU558702 | KU558702 |
| KX550281 | KX550281 | KX550281 |
| KY019623 | KY019623 | KY019623 |
| KU982966 | KU982966 | KU982966 |
| KU982968 | KU982968 | KU982968 |
| KU569509 | KU569509 | KU569509 |
| KR265766 | KR265766 | KR265766 |
| KR265831 | KR265831 | KR265831 |
| LC063812 | LC063812 | LC063812 |
| LC063815 | LC063815 | LC063815 |
| LC063816 | LC063816 | LC063816 |
| LC063832 | LC063832 | LC063832 |
| LC063840 | LC063840 | LC063840 |
| KP728470 | KP728470 | KP728470 |
| KR610993 | KR610993 | KR610993 |
| KR011756 | KR011756 | KR011756 |
| KM609210 | KM609210 | KM609210 |
| KM609211 | KM609211 | KM609211 |
| LM645057 | LM645057 | LM645057 |
| KJ960179 | KJ960179 | KJ960179 |
|  | KJ645702 | KJ645702 |
|  | KJ645704 | KJ645704 |
| KC109141 | KC109141 | KC109141 |
| JX647847 | JX647847 | JX647847 |
| JN547228 | JN547228 | JN547228 |
| GU937797 | GU937797 | GU937797 |
|  | EF185992 |  |


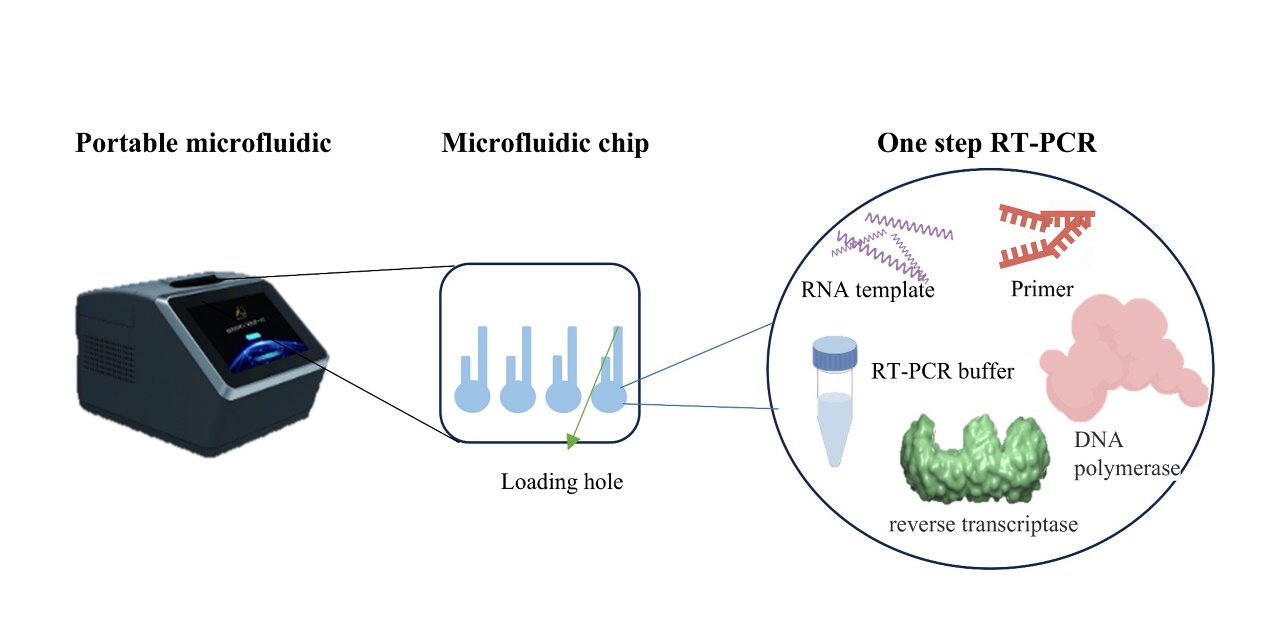


Supplement Figure 1 POC-NAD based on one-step RT-PCR method and microfluidic technology


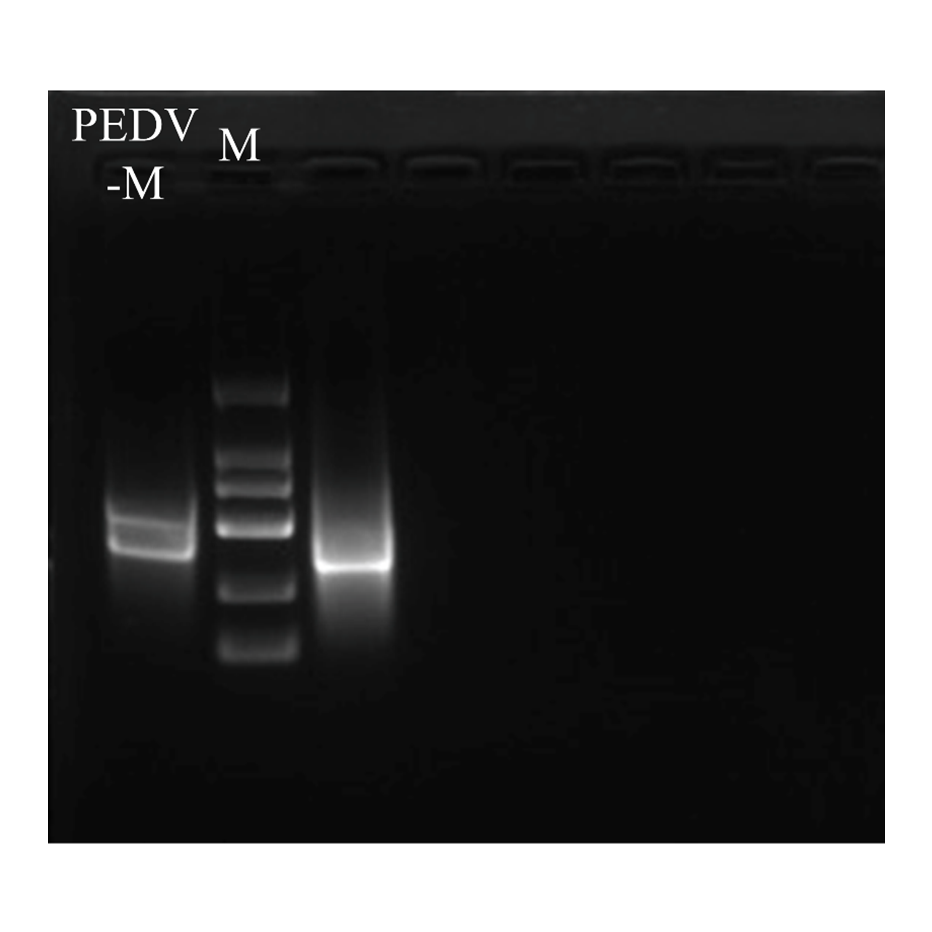


Supplement Figure 2 PEDV-M gene was amplified using PEDV cDNA (CV777) as a template, and plasmid was constructed using the pEASY-Blunt cloning kit. Transcribed in vitro using the T7 High Yield RNA Transcription Kit (Vazyme) following the manufacturer's protocol.

Supplement Table2 Background and sample Types of 19 swine clinical samples.

| Sample No. | Background | Types | Sample No. | Background | Types |
| --- | --- | --- | --- | --- | --- |
| 1 | PEDV+ | Intestinal tissue | 2 | PEDV+ | Intestinal tissue |
| 3 | PEDV+ | Intestinal tissue | 4 | PEDV+ | Intestinal tissue |
| 5 | PEDV+ | Intestinal tissue | 6 | PEDV+ | Intestinal tissue |
| 7 | - | Intestinal tissue | 8 | PEDV+ | Intestinal tissue |
| 9 | PEDV+ | Intestinal tissue | 10 | PEDV+ | Intestinal tissue |
| 11 | PEDV+ | Intestinal tissue | 12 | PEDV+ | Intestinal tissue |
| 13 | PEDV+ | Intestinal tissue | 14 | PEDV+ | Intestinal tissue |
| 15 | PEDV+ | Intestinal tissue | 16 | PEDV+ | Intestinal tissue |
| 17 | - | Intestinal tissue | 18 | - | Intestinal tissue |
| 19 | - | Intestinal tissue |  |  |  |
